# Supplementary material for: PLOS Medicine 2015 Reviewer Thank You
Source: PLoS Med. 2016 Feb 23;13(2):e1001983. doi: 10.1371/journal.pmed.1001983 (PMC4764359; doi:10.1371/journal.pmed.1001983)
Supplement: S1 Reviewer List — (PDF) [file pmed.1001983.s001.pdf]

PLOS | MEDICINE

PLOS Medicine would like to thank all those who reviewed on behalf of the journal in 2015:

Takeru Abe
Carla Abouzahr
Adine Adonis
Kathleen Akgun
Harold Alderman
Jared Aldstadt
G. Caleb Alexander
Meredith Alger
Marjan Alssema
Galit Alter
Yuri Amirkhanian
Joseph Amon
Frank Anania
Alexander Anderson
Ian Anderson
Peter Andrews
Oskar Angerås
Josh Angrist
Nicholas Anstey
Richard Anthony
John Aponte
Chris Ardern
Ramy Arnaout
Sandra Arnold
Mauricio Avendano
Hatem Azim
Andrew Azman
Leonard Bacharier
Marc Baguelin
Robert Bailey
George Bakris
Paul Bangirana
David Bangsberg
Raveendhara Bannuru
Sergio Baranzini
Michelangela Barbieri
Virginia Barbour
Peter Barnes
Scott Barnhart
Till Bärnighausen
Hilda Bastian
Paul Batalden
Robert Beaglehole
M. Flint Beal
Jasper Been
James Beeson
Marcel Behr
Philip Bejon
Eran Bendavid
Charles Bennett
Sara Bennett
James Berkley
Seth Berkowitz
Sophie Bertrand
Achuyt Bhattarai
Raj Bhopal
Peter Biberthaler
Arlene Bierman
Elisabeth Binder
Zeno Bisoffi
Anders Björkman
Sean Blackwell
Tony Blakely
John Blandford
Catherine Blish
Jason Block
Michael Boivin
Amy Bonomi
Heather Boonstra
Martin Bootsma
Luciana Borio
Xavier Bosch
Hervé Bourhy
Teun Bousema
Jack Bowden
Fran Boyle
Tom Boyles
Soren Brage
Carol Brayne
James Brenton
Michael Bretthauer
Grania Brigden
John Britton
Shannon Brownlee
Craig Bryan
Jennifer Bryce
Stephen Burgess
Freddie Bwanga
Peter Byass
Tim Byers
Helen Bygrave
Christopher Callahan
Gideon Caplan
Daniel Carlat
Andrew Carr
Alessandro Cassini
Timothy Caulfield
Jean-Marc Cavaillon
Lucy Chappell
Yun-Jiu Cheng
Lumbwe Chola
Gerardo Chowell
Kathryn Chu
Lauren Cipriano
Aileen Clarke
Hance Clarke
James Cleary
Archie Clements
Myron Cohen
Miriam Colombo
Margaret Conner
Jason Connor
Ronan Conroy
Ben Cooper
Tony Cornford
Catherine Cornu
Jane Costello
Scott Cousins
Benjamin Cowling
Sondra Crosby
Ron Dagan
Darren Dahly
Ann Daly
Christof Dame
Goodarz Danaei
Nick Daneman
Karen Daniels
George Davey Smith
Neil Davies

Kevin De Cock
Harry de Koning
Douglas Delahanty
Nikos Demiris
Sachin Desai
Delan Devakumar
Puneet Dewan
Michael Dewey
Monica Dhakar
Kumar Dharmarajan
Mehul Dhorda
William Dixon
Tanya Doherty
Christl Donnelly
Peter Doshi
Delanyo Dovlo
John Drake
Alison Drake
Tom Drake
Colin Drummond
Mignon Duplessis
Philip Eckhoff
Tim Eckmanns
Costanza Emanueli
Jonathan Emberson
Sean Emery
Ingunn Engebretsen
Ralph Epaud
Susanna Esposito
David Evans
Carlton Evans
Rick Fairhurst
Victoria Fan
Maha Farhat
Nicola Fawcett
Seena Fazel
Neelam Feachem
Neil Ferguson
Rashida Ferrand
Guido Filler
Adam Finn
Stefan Flasche
Peter Flom
Nathan Ford
Lui Forni
Freya Fowkes
Katherine Fowler
Timothy Frayling
Caroline Free
Lynn Freedman
Matthew Freeman
Tim Friede
Thomas Friedrich
Jennifer Furin
Moses Galukande
Sylvain Gandon
Paul Garner
Tini Garske
Coral Gartner
Ruowen Ge
Abdi Ali Gele
Murat Genc
Elvin Geng
Patrick Gérardin
Patrick Gerland
Abdul Ghafur
Cyrus Ghajar
Davina Ghersi
Keon Gilbert
Dean Giustini
Melissa Gladstone
Stanton Glantz
Amanda Glassman
Richard Glassock
David Goldblatt
Robert Goldenberg
Jeremy Goldhaber-Fiebert
Pamina Gorbach
Manuel Graeber
Trevor Graham
Rebecca Grais
Raquel Granell
Robert Grange
Trisha Greenhalgh
Nicola Greenlaw
Brian Greenwood
Jamie Griffin
Elena Grigorenko
Carlos Grijalva
Timo Grimmer
William Grobman
Scott Grosse
Guangwei Li
Tongsheng Guo
Emily Gurley
David Gurwitz
Else Guthrie
Jessica Haberer
Pranabashis Haldar
William Haley
Robert Hall
Wayne Hall
Timothy Hallett
Claudia Hanson
Diane Harper
Karin Hatzold
Phillipa J. Hay
Richard Haynes
David Healy
Sean Heffron
Leonhard Held
Marco Helder
Marie Helleberg
Orjan Hemstrom
David Henry
Hector Hernandez-Vargas
Pedro Herrera
Andrew Herxheimer
Bradford Hesse
James Hicks
Suzanne Hill
David Himmelstein
David Hipgrave
John Hoddinott
Stephen Hodgins
T. Deirdre Hollingsworth
Lars Holmberg
Wendy Holmes
Jan Hontelez
Robert Hoover
Nils Hoppe
Richard Hornung
C. Robert Horsburgh
Erik Hoyer
Frank Hu
Stefan Huber-Wagner
Megan Huchko
Jean Humphrey
Paul Hunter
Andrew Hutchings
Lars Hviid
Emily Hyle
Fumiaki Imamura
Egeruan Imoukhuede

Chandra Jackson
Anders Jacobsen Skanderup
Karen Jacobson
Laura James
Robert Jeffery
Aksel Jensen
Christie Jeon
James Johnson
Markus Jokela
Hayley Jones
Richard Jones
Louisa Jorm
Elmar Joura
Theodore Joyce
Min Jun
Jon Jureidini
James Kahn
Joan Kalyango
Adam Kamradt-Scott
Steve Kanters
Amy Kapczynski
John Kaplan
George Karam
Stephanie Karst
Harin Karunajeewa
Ingrid Katz
Jay Kaufman
Anne Kavanagh
Brendan Keating
Alexander Kekulé
Mirjam-Colette Kempf
Thomas Kenyon
George Kephart
Marko Kerac
Aaron Kesselheim
Saira Khowaja
Peter Kilmarx
Lesley King
Michael King
Sanjay Kinra
Avinash Kishore
Florian Klein
Eili Klein
Pete Kolsky
Franz König
James Koopman
Frederick Korley
Marion Koso-Thomas
Anita Koushik
Roger Kouyos
Katy Kozhimannil
Katharina Kranzer
Ronald Krauss
Margaret Kruk
Jim Kublin
Jayashri Kulkarni
Michael Kuo
Daniel Kuritzkes
Niklaus Labhardt
Nicos Labropoulos
Hiddo Lambers Heerspink
Claudio Lanata
Bruce Lanphear
Sean Lawler
Michael Lebens
James Lecheminant
Won Jin Lee
Kelley Lee
Richard Lehman
Maria Lemos
Justin Lessler
Michael Levin
Kristina Lewis
Joel Lexchin
Stefan Lhachimi
Qingqin Li
Benjamin Linas
Richard Lindley
Steve Lindow
Lingzhong Xu
Sebastien Lion
Marc Lipsitch
Suzanne Lloyd
Stacy Loeb
Ira Longini
Julie A. Lovegrove
Stephen Luby
Sebastian Lucas
Peter Lurie
Ronald Ma
Michael Maciosek
Ulrich Mansmann
Riccardo Marioni
Jeanne Marrazzo
Marta Marthas
Natasha Martin
Soeren Mattke
Richard Maude
Deborah Mc Farland
Alex McConnachie
James McCormack
Peter McCulloch
Ian McDowell
Grant McFadden
Patricia McGettigan
Sean McGuigan
Leemon McHenry
Peter McIntyre
Martin McKee
Mary-Louise McLaws
Andrew McMichael
Peter McMinn
Ravindra Mehta
David Menkes
John Metcalfe
Philippa Middleton
Steven Miles
Elizabeth Miller
Grant Miller
Jeffrey Miller
Edward Mills
Geltrude Mingrone
Barbara Mintzes
Masaaki Miyata
Erika Moen
Lynne Mofenson
Sulma Mohammed
Ali Mokdad
Igor Mokrousov
Joanna Moncrieff
Dominic Montagu
Federica Montanaro
Suerie Moon
Karel Moons
Siobhan Mor
Judith Mueller
Satinath Mukhopadhyay
Luke Mullany
Bertram Müller-Myhsok
Heather Murray
Senthil Muthuswamy
Paul Myles
Jean Nachega
Arijit Nandi

Steven Narod
Bruce Neal
Paul Newcombe
Marie-Louise Newell
Elizabeth Newnham
Olivier Neyrolles
Daan Nieboer
Michael Niederman
Jacek Niedziela
Stefan Niemann
John Nkengasong
Teresa Norat
Thomas Novotny
Martin Nowak
Carole Ober
Paul O'Brien
James O'Connor
Shuji Ogino
Tim Olde Hartman
Bolajoko Olusanya
Steven Opal
David Osrin
Ingegerd Östman-Smith
Rosalie Pacula
Madhukar Pai
Neil Pakenham-Walsh
Smita Pakhale
Suetonia Palmer
Andrew Papachristos
Neehar Parikh
Anushka Patel
Angel Paternina-Caicedo
Mical Paul
David Peiris
Jill Pell
Matthew Penn
Loveday Penn-Kekana
Roy Perlis
Navindra Persaud
Sarah Peters
Ulrike Peters
Stefan Peterson
Genevieve Pham-Kanter
Amy Pickering
Richard Pitman
Harold Pollack
Alan Poots
Donald Poretz
Andrew Prendergast
Ric Price
Ian B. Puddey
Marja Puurunen
Michael Ramharter
Fahad Razak
K. Srinath Reddy
Donald Redelmeier
Bin Ren
Frank Riede
Steven Riley
David Rimm
Giovanni Ristori
Alison Ritter
Bjarne Robberstad
Claire Roberts
Lucy Robertson
Pamela Robey
Bryan Rodgers
Michael Roerecke
Stephen Rogerson
Pejman Rohani
Susannah Rose
Joseph Ross
Lionel Rostaing
Kenneth Rothman
Edward Ryan
Kerry-Anne Rye
David Sack
Mary Sano
Stephanie Sansom
Amir Sariaslan
Ameet Sarpatwari
Minnie Sarwal
Oliver Schildgen
Kevin Schulman
Roland Schwarz
Nicholas Selby
Hilary Seligman
Dinesh Selvarajah
Srijan Sen
Jaime Sepulveda-Amor
Kwonjune Seung
Nilay Shah
Nuala Sheehan
Jeremy Shiffman
Elisa Sicuri
Arjumand Siddiqi
Mark Siedner
Mervyn Singer
Abhijeet Singh
Valérie Siroux
Tami Skoff
Jolene Skordis-Worrall
Mary Slack
Thomas Smith
Elizabeth Smith
Gordon Smith
Stephanie Smith
Peter Smith
Laramie Smith
Lucy Smith Paintain
Jonathan Snowden
Maria Pia Sormani
Glen Spielmans
Devi Sridhar
Charles Ssonko
Charles Stanley
Lincoln Stein
David Stephens
Peter Sterk
Ewout Steyerberg
Ute Stroeher
David Stuckler
David Studdert
S.V. Subramanian
Jonathan Sugimoto
Jeremy Sussman
Amitabh Suthar
Timothy Sweeney
Rohan Sweeney
Susan Swindells
Maarten Taal
Shahrad Taheri
Kristina Talbert-Slagle
Ambrose Talisuna
Bee Kang Tan
Navdeep Tangri
Peter Tanuseputro
Andrew Tatem
Madhav Thambisetty
Harsha Thirumurthy
Rebecca Thornton
Roland Thorpe Jr.
Mark Tomlinson
Oyewale Tomori

Alexandra Trkola
Alexander Tsai
James Tumwine
Janet Turan
Thorkild Tylleskär
Jessica Tyrrell
Leanne Unicomb
Patrick Vallance
Wim van Biesen
R.J. van Bommel
Janneke van de Wijgert
M. Flint van den Heuvel
Jacques van der Gaag
Wim van Harten
Michel van Herp
Anne van Kempen
Richard van Zyl-Smit
Effy Vayena
Atheendar Venkataramani
Willem Venter
Stephane Verguet
Sten Vermund
Cecile Viboud
Sandeep Vijan
Jean-Louis Vincent
Julian Vivian
Benjamin Voight
Jimmy Volmink
Fernando Volpe
Arnold von Eckardstein
Lorenz von Seidlein
Kranti Vora
Theo Vos
Bradley Wagner
Peter Waiswa
Levi Waldron
Robert Wallis
Xiao-Fan Wang
Xuan Yi Wang
Rebecca Warburton
Nicholas Wareham
James Wason
Catherine Weil-Olivier
Daniel Weinberger
Vivian Welch
Lori West
Gillian Whalley
Paul Whelton
Peter White
Harvey Whiteford
George Whitesides
Christopher Whitty
Beate Wieseler
Sarah Wild
Thomas Williams
Brian Williams
David Wilson
Sidney Wolfe
Hector Wong
Charles Woodrow
Mark Woodward
Jim Wright
Joseph Wu
William Wunner
Kiran Yanamandra
Tse-Chuan Yang
Patsy Yates
Jessica Yeats
Aisha Yousafzai
Huang Yu
Jonathan Zelner
Jian-Hong Zhong
Cathy Zimmerman
Susan Zolla-Pazner
Thomas Zoller
